# Gut in Tube—Continuous Measurement of Metabolic Crosstalk between Cell Populations in Heterogeneous Samples by NMR Imaging

**DOI:** 10.1021/acs.analchem.4c05156

**Published:** 2025-02-27

**Authors:** Todor T. Koev, Hou Hei Chung, Caitlin Wright, Evie Banister, Stephen D. Robinson, Matthew Wallace

**Affiliations:** † School of Chemistry, Pharmacy and Pharmacology, University of East Anglia, Norwich Research Park, Norwich NR4 7TJ, U.K.; ‡ School of Pharmacy, University of Nottingham, Nottingham NG7 2RD, U.K.; § School of Biological Sciences, University of Manchester, Manchester M13 9PL, U.K.; ∥ School of Biological Sciences, University of East Anglia, Norwich Research Park, Norwich NR4 7TJ, U.K.; ⊥ Food, Microbiome and Health, Quadram Institute Bioscience, Norwich Research Park, Norwich NR4 7UQ, U.K.

## Abstract

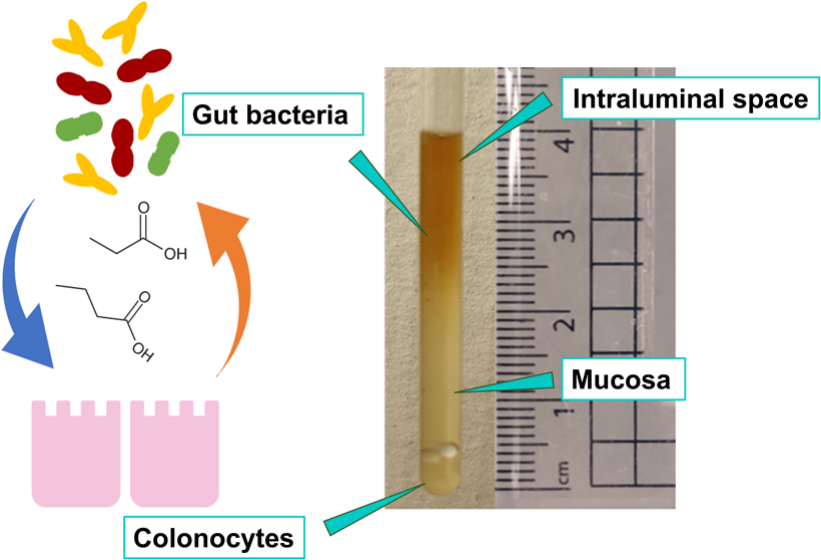

In complex living systems, such as the human gut, the interplay between the multiple cell types present is governed by the exchange of small molecule metabolites. However, at present, we lack techniques capable of monitoring this crosstalk in real time and with spatial resolution. Here, we present a model of the human gut in a 5 mm NMR tube that accounts for the intraluminal, mucosal, and colonocyte spaces. Cells are cultured in different spatial regions enabling metabolites, changes in pH, and the effects of exogenous molecules to be monitored exclusively using localized NMR techniques. Our model represents a high-throughput, readily available, and widely applicable approach to the study of living systems with multiple cell types on a molecular level. We used our model to explore the interplay between gut bacteria and colonocytes in the human large intestine and study the molecular concentration gradients naturally present in these systems. Such studies could help shed light on the crucial role played by the gut microbiota in maintaining gut homeostasis, modulating immune responses, metabolizing nutrients, and regulating host physiology.

## Introduction

Living systems from the gut to biofilms are inherently heterogeneous and feature multiple cell types. The interactions between these cells are governed by the exchange of small molecules along concentration and pH gradients. However, current approaches to studying these dynamic systems either lack spatial resolution (NMR) or are unable to reveal the identities or concentrations of the metabolites being exchanged. Here, we show how the *z*-axis spatial resolution afforded by the field gradient coils of conventional high-resolution NMR probes enables the construction of a vertically oriented model of the human gut directly in a 5 mm NMR tube. Our model features immobilized colonocytes at the bottom of the tube, a mucosal layer, and planktonic commensal bacteria above. Our approach represents a broad-spectrum strategy to monitor molecular exchange in complex living systems featuring multiple cell types using common analytical equipment.

The human gut microbiome has emerged as a powerful determinant of host health and has been associated with several important comorbidity factors and disease states including obesity, metabolic syndrome, bowel cancer, and neurodegenerative disorders such as Alzheimer’s and Parkinson’s disease.^1^ As key players in the human large intestinal environment, the interplay among the gut microbiota, intestinal mucosa, and colonocytes has been shown to play an important role in modulating host immunity and conferring resistance to infection.^2−4^ Previous works have demonstrated enterocytes’ utilization of short-chain fatty acids (SCFAs) produced by gut microbes.^5−7^ However, not much is known about the multidirectional exchange of small molecules among colonocytes, commensal bacteria, and the gut mucosa across different spatial regions of the large intestine. We currently lack high-throughput models and methods able to study these processes at a molecular level while also reproducing key physiological traits of the human large intestine.^5^ Currently existing techniques for probing the exchange between colonocytes and gut microbial species rely on specialist equipment and coculture methods that can be difficult to set up. Examples include gut-microbiome physiomimetic platforms,^2^ organ-on-chip technologies,^8^ and diffusion chambers.^9^ These techniques do not provide key molecular-level information, such as real-time pH measurement and SCFA profiling—important parameters for the detection of metabolic pathogenesis.^3,4^

Chemical shift imaging nuclear magnetic resonance (CSI NMR) allows for the continuous quantitative spatial mapping of complex mixtures and heterogeneous samples on a molecular level.^10,11^ Here, we create a model of the human large intestine directly in a standard NMR tube, featuring vertically oriented luminal, mucosal, and colonocyte regions. Our model combines colonocytes of four different lineages together in three-dimensional (3D) culture (Caco-2, T84, HT29, and SW620)—a mixture of mucus-secreting and absorptive enterocytes/colonocytes—which represent the major cell types in the large intestine.^12^ The model features both loosely and tightly bound mucosal layers^13,14^ and is inoculated using fecal samples from a healthy human. We apply CSI techniques^15,16^ to probe changes in pH in real time using standard, widely available NMR equipment, and in-house-written automation scripts for rapid data processing.^17^ Furthermore, our method allows for the spatially resolved quantification of key metabolites such as SCFAs at a 0.5 mm spatial resolution and the assessment of how they mediate crosstalk between commensal bacteria and colonocytes. Finally, we show how our methodology can be applied to probe drug transit through a model of the intestinal luminal space and mucosa—key for optimal drug development and delivery to physiologically relevant regions such as the distal parts of the human gastrointestinal tract.^18^

## Materials and Methods

### Materials

All compounds, reagents, and cell culture materials were obtained from Merck (formerly Sigma-Aldrich, Darmstadt, Germany), and Sarstedt (Sarstedt AG & Co., KG, Nümbrecht, Germany) unless otherwise specified.

### Methods

#### Cell Culture

Complete cell culture media was prepared by supplementing high-glucose Dulbecco’s modified Eagle‘s media (DMEM, 4.5 g/L glucose, sodium pyruvate CAS D7777) with bovine calf serum (BCS, 20% v/v, CAS 12138C), nonessential amino acids (NEAA, 1.0% v/v, CAS M7145), l-glutamine (1.0% v/v, CAS G8540), and penicillin/streptomycin (P/S, 1.0%, CAS P4333).

Individual cell lines (Caco-2, HT29, T84, and SW620) were grown from frozen stocks, prepared at *ca*. 1 million/mL. Cells were seeded in a T-75 tissue culture flask, passaged once, and matured to *ca*. 10 million/mL in a T-175 flask in complete media. Cells were rinsed twice with Ca^2+^/Mg^2+^-free PBS (25 mL, CAS D8537), trypsinized, the trypsin deactivated with P/S-free complete media, all four lineages mixed in a 1:1:1:1 ratio, pelleted (300 rcf, 5 min), and resuspended in alginate (sodium alginate, CAS 9005–38–3, 1% w/v in P/S-free complete media). The cell suspension was extruded through a 200 μL pipet tip dropwise into cold CaCl_2_ (0.3 M in phosphate-buffered saline, PBS, CAS D8662, 15 °C) under stirring (100 r.p.m.). The cell spheres (Figures S1 and S2, Supporting Information (SI)) were removed from the CaCl_2_ solution, washed, and stored in P/S-free complete media before further use.

#### Model Setup

Synthetic mucus was prepared similar to previous works.^19,20^ Briefly, it was reconstituted from porcine gastric mucus (PGM, CAS M2378) in P/S-free complete media (4% w/v) and left to stir for 2 h (room temperature, 100 r.p.m.). 4-Arm PEG-thiol (PEG-4SH, Laysan Bio Inc., 174–47), a cross-linking agent, was prepared in P/S-free complete media (4% w/v) and mixed at equal volumes with the PGM solution, resulting in a final 2% w/v of both PGM and PEG-4SH. Sodium acetate trihydrate (CAS 6131–90–4), disodium maleate (CAS 25880–69–7), and 3-(trimethylsilyl)-1-propanesulfonic acid sodium salt (DSS, CAS 2039–96–5) were added to the suspension to a final concentration of 5 mM of each compound.

Commensal inoculum was prepared by diluting a fresh fecal sample in P/S-free complete media (30% w/v), which had been left in an anaerobic cabinet overnight at 37 °C. Acetate, maleate, and DSS were anaerobically added at a final concentration of 5 mM. The final suspension was mixed with equal parts of the synthetic mucus (300 μL each), vortexed for 30 s, and incubated at 37 °C for 1 h prior to inoculating the model.

**Figure 1:**
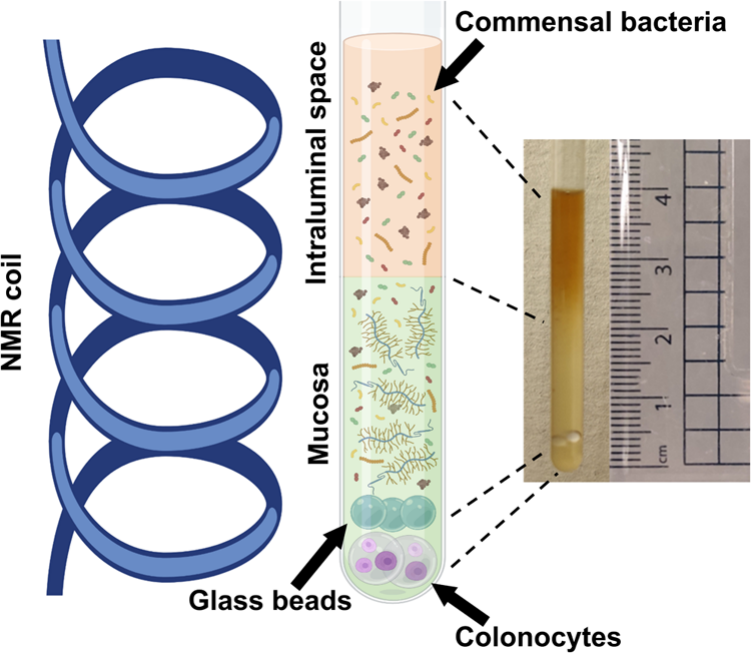
Illustration of the sample setup against a photo of the actual sample with a ruler showing the true depth of the NMR sample. A cartoon tube superimposed against the dimensions of the NMR radiofrequency coil showcasing the active volume of the spectrometer. Partially created in BioRender. Koev, T. (2025) https://BioRender.com/j03d772.

Three cell-containing spheres were placed at the bottom of a screw-cap NMR tube (Wilmad, Z271942), followed by 3 glass beads (2.0 mm diameter) to prevent the cell-containing spheres from floating on the generation of CO_2_, followed by pipetting 600 μL of the inoculated mucosa (Figure 1). The NMR tube was degassed with CO_2_ through the septum for 1 min, before placing it in the NMR spectrometer, preset at 37 °C, and left to settle for 15 min before starting the experiments to allow for the mucosal and intraluminal layers to settle and avoid any immediate mixing effects.

#### Chemical Shift Imaging NMR

All CSI NMR experiments were performed off-lock at 310 K on a Bruker 500 MHz AVANCE NEO spectrometer, operating at a ^1^H frequency of 499.31 MHz. ^1^H CSI experiments were acquired using a gradient-phase-encoding sequence based on the work of Trigo-Mouriño et al.,^21^ featuring a double echo excitation sculpting (Bruker library, *zgesgppe*) as a solvent-suppression component, using 4 ms Gaussian inversion pulses. The phase-encoding gradient pulse (172 μs) was in the form of a smoothed square, ramped from −18.8 to 18.8 G/cm in 64 steps. ^1^H *rf* pulse was 8.43 μs, and a total of 4 scans were acquired at each gradient increment, with 16 dummy scans, an acquisition time of 2 s, and a recycle delay of 4 s. A spoil gradient pulse (600 μs, 26 G/cm) was applied at the end of the acquisition to destroy any remaining magnetization. The vertical range of the experiment was set to 2.99 cm, giving a theoretical spatial resolution of *ca*. 0.6 mm. A total of 30 experiments were carried out with a 30 min delay between two consecutive experiments, covering a total experimental window of 24 h per sample.

To determine p*K*_a_, δ_H_, and δ_L_ for acetate and maleate (eq 1) at 310 K, buffer solutions^22^ were prepared in *dd*H_2_O containing 1.0 mM pyrazine, DMSO, 0.4 mM DSS, and 0.2 mM each of disodium maleate, acetate, and methylphosphonate at pH 4.025 (50 mM potassium hydrogen phthalate), pH 6.84 (25 mM K_2_HPO_4_ and 25 mM NaH_2_PO_4_), and pH < 1.5 (50 mM HCl). The low concentrations of these indicator bases do not significantly affect the pH of the buffers. A multicomponent buffer solution (pH > 10) was also prepared containing 10 mM Na_2_HPO_4_, NaOB(OH)_2_, sodium acetate-*d*_3_, tris-*d*_11_, 20 mM Na_2_CO_3_, 4.0 mM disodium methylphosphonate, glycinate, formate, 2.0 mM disodium maleate, acetate, 1.0 mM 4-(2-hydroxyethyl)piperazine-1-ethanesulfonic acid (Hepes), 1.0 mM pyrazine, DMSO, and 0.4 mM DSS. δ_L_ of methylphosphonate, maleate, acetate, formate, and Hepes were measured directly from this solution (pH > 10). δ_H_ values of acetate and formate were measured in 50 mM HCl. The p*K*_a_ of formic acid and acetic acid were determined from their chemical shifts in the potassium hydrogen phthalate buffer by reversing eq 1 and inserting the chemical shifts (δ_obs_) measured in this buffer (2.0482 and 8.3877 ppm for acetate and formate, respectively).

The multicomponent buffer solution was carefully layered on top of 2.0 mg of oxalic acid in a 5.0 mm NMR tube, following our published procedure.^15,17^ The sample was then placed in a Bruker 800 MHz Avance III spectrometer at 310 K for 14 h for a pH gradient to develop, after which a 64-point CSI experiment was carried out. δ_H_ of methylphosphonate was taken as the observed chemical shift in a row of the CSI data set with a pH of 4.7, as judged from the chemical shift of acetate. The p*K*_a_ of methylphosphonate was then obtained by reversing eq 1 and using δ_obs_ measured in phosphate buffer (pH 6.84). The pH of the solution was determined along the pH gradient from the chemical shifts of acetate and methylphosphonate, allowing δ_H_, δ_L_, and p*K*_a_ of maleate and Hepes to be determined by fitting their observed chemical shift as a function of pH to eq 1 (Figure S3, SI)
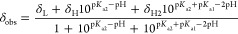
1

where δ_H2_ is the limiting chemical shift of the indicator in its doubly protonated state; however, this second protonation step (p*K*_a2_) cannot be accessed with our pH gradient. Nevertheless, only the first protonation step (p*K*_a1_) needs to be considered in our experiments as pH > 4 in all cases. Finally, the “mixed” p*K*_a_ in the media used for cell culture (ionic strength, *I* = 0.174 M) was calculated from the “mixed” p*K*_a_ (p*K*_a_*), measured as described in the above paragraph and as obtained via fitting to eq 1, by first correcting it to the thermodynamic p*K*_a_ (p*K*_a,0_) using eq 2(23)
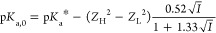
2

The dielectric constant of the medium at 310 K is assumed equal to 74.31 and is used to calculate the Debye–Hückel terms of 0.52 and 1.33, assuming an ionic radius of 4 Å.^24,25^*Z*_H_ and *Z*_L_ are the charges of the indicator species in their protonated and deprotonated states, respectively. Limiting chemical shifts and p*K*_a_ values for all indicators are provided relative to DSS at 0 ppm (Table S1, SI). These limiting values can be related to DMSO or pyrazine as chemical shift references, as these compounds are invariant with pH (Figure S3c), averaging 2.7141 ppm (DMSO) and 8.6434 ppm (pyrazine). The pH was calculated by taking the sensitivity-weighted average of the pH reported by acetate and maleate, as described in our previous work.^15,17^

#### Data Processing

All ^1^H CSI data sets were processed with an exponential line broadening factor of 3.0 Hz. CSI data sets were processed in phase-sensitive mode, with phase, baseline correction, and chemical shift referencing to DSS (0.0 ppm) performed automatically using in-house-written scripts.^17^

#### Peptide Synthesis

Synthesis of *p-*F-Phe-Ala-His-Trp was synthesized by Fmoc-solid phase peptide synthesis using appropriately protected Fmoc-amino acids. Rink amide 4-methylbenzhydryl amine (MBHA) resin (*ca*. 100 mg) was swelled for 30 min in *N*,*N*-dimethylformamide (DMF). The resin was deprotected with piperidine in DMF (20%, 20 min) twice, followed by washing three times with DMF. The coupling of Fmoc-protected amino acids was completed with *N*,*N*,*N*′,*N*′-tetramethyl-*O*-(1*H*-benzotriazol-1-yl)uranium hexafluorophosphate/1-hydroxybenzotriazole hydrate (HBTU/HOBt, 4 equiv) and diisopropylethylamine (DIPEA, 8 equiv) and shaken (60 rpm, 45 min). Each peptide coupling was repeated, and then the resin was washed three times with DMF. After each peptide coupling, the growing peptide chain was deprotected with piperidine. Cleavage of the peptide from the resin was completed in 95:2.5:2.5 trifluoroacetic acid:triisopropylsilane:H_2_O with shaking (60 r.p.m., 3 h). The resulting solution was evaporated to dryness and washed with cold ether. The resulting peptide was purified by preparative RP-HPLC, and then the purity was confirmed by analytical RP-HPLC, collecting the peptide as a yellow solid. RP-HPLC retention time: 12.41 min. MALDI-ToF (*m*/*z*): calculated 577.6636; found: 577.6162.

#### Experimental and Predicted Peptide Diffusion through Colon Model

The diffusion of the tetrapeptide was measured experimentally by measuring its concentration along the entire depth of the NMR tube 6 and 12 h after the beginning of the experiment. To obtain its experimental diffusion coefficient, the peptide’s diffusion was iteratively fitted (eq 3) using the Solver Add-in package for MS Excel until a maximum *R*^2^ value was obtained (*R*^2^ = 0.993 and 0.996, Figures S4 and S5, SI)
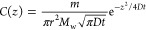
3

where *C* is the concentration of the peptide at each vertical position (*z*), m is the mass of the peptide, *r* is the radius of the NMR tube, *M*_w_ is the molecular weight of the peptide, *D* is its self-diffusion coefficient, and *t* is the time of the experiment since preparation.

The self-diffusion coefficient of the peptide was calculated as 4.8 × 10^–10^ m^2^ s^–1^ (Figures S4 and S5, SI) using the Stokes–Einstein Gierer–Wirtz estimation (SEGWE) presented by Evans et al.^26^

#### Cell Viability Assay

The effect of the peptide on cell viability was measured using a cell proliferation kit (CAS 11465007001). 3-[4,5-Dimethylthiazole-2-yl]-2,5-diphenyltetrazolium bromide (MTT) was solubilized in PBS (5 mg/mL) and filter-sterilized (0.22-μm). Cocultures of colonocytes were grown in phenol red-free complete media in 24-well plates to *ca*. 50% confluency. The medium was removed, cells were washed twice with PBS, and equal volumes of serum-free medium and MTT solution were added to each well. The fluorinated peptide was added to half of the wells (1.6 mg/mL). The cells were incubated (37 °C, 3 h), followed by the addition of MTT solvent (4 mM HCl, 0.1% (w/v) NP40 in isopropanol). The plate was wrapped in aluminum foil and placed on an orbital shaker (60 rpm, 15 min). After shaking, the solution in each well was resuspended several times with a pipet to ensure full solubilization of the resulting formazan. The absorbance (OD_590_) in each well was read, and % viability was calculated against the control (Figure S6, SI).

#### Bacterial Viability Assay

2 mL aliquots of phenol red- and P/S-free media were placed in each well of a 24-well plate and inoculated with fresh fecal inoculum (100 μL of fresh fecal inoculum diluted in phenol red- and P/S-free media, 30% w/v). The fluorinated peptide was added to half of the wells (1.6 mg/mL). Bacteria were incubated anaerobically (37 °C, 24 h). The absorbance (OD600) in each well was measured, and % viability was calculated against the control (Figure S7, SI).

#### Microscopy

Cocultures of the four distinct cell lineages were grown in coverslip dishes (Mattek, P35G-1.5–10-C) until *ca*. 90% confluence. Cells were washed (Ca^2+^/Mg^2+^-free PBS) twice and fixed in *p*-formaldehyde (4%, *p*FA, CAS 1.00496) for 10 min. The fixative was washed twice more. Sites of nonspecific binding were blocked with BCS (10% in PBS, 37 °C, and 1 h). Primary antibody (anti-MUC2, rabbit monoclonal, AbCam, ab272692) was added as a 1:10000 dilution in blocking solution, along with Hoechst 33342 (ThermoFisher, H3570, 1 mg/mL), and incubated (4 °C, overnight). Cells were washed twice with cold PBS, followed by the addition of the secondary antibody (antirabbit, donkey conjugated with Alexa Fluor 647) at 1:10000 in PBS and incubated (4 °C, overnight). Nonbound antibodies were washed with PBS 3 times. Samples were visualized on a Zeiss Axio Observer 7 inverted microscope at 20-, 40-, and 60-fold magnifications (Figure S8).

#### Statistical Analyses

The statistical significance (*p*-value) was determined through a combination of unpaired *t* test and two-way ANOVA, using GraphPad Prism 9.0.0, based on a minimum of three replicates, where ns *p* > 0.05, *p < 0.05, and **p < 0.01, ***p < 0.001.

## Results and Discussion

**Figure 2:**
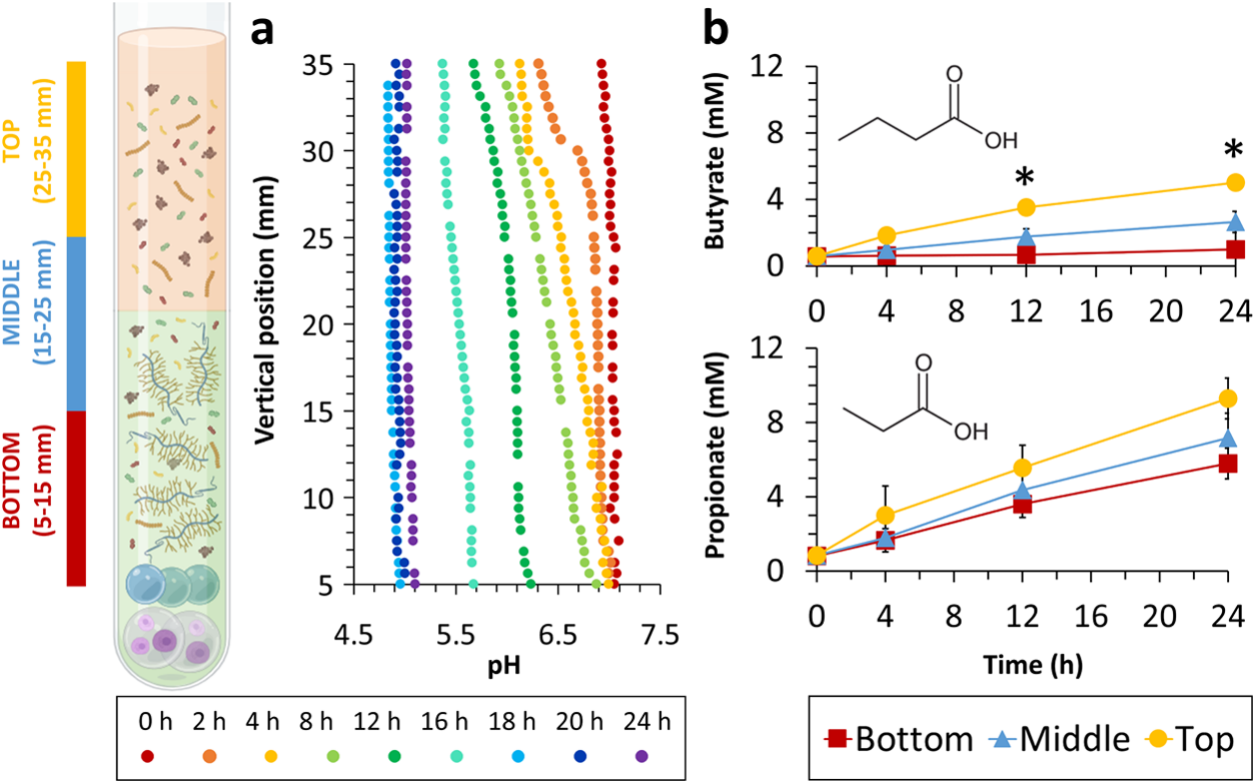
Spatially resolved pH change across our model over 24 h (a). Concentration of butyrate and propionate over 24 h across the top (yellow circle, 25–35 mm), middle (blue triangle, 15–25 mm), and bottom (red square, 5–15 mm) of the NMR tube (b), *p < 0.05, *n* = 3. Partially created in BioRender. Koev, T. (2025) https://BioRender.com/j03d772.

Using CSI techniques, we record changes in pH via the ^1^H chemical shifts of acetate (p*K*_a_ = 4.58) and maleate (p*K*_a_ = 5.93) in 8 min experiments, allowing continuous measurements over 24 h. Since on an NMR time scale, the observed chemical shift, δ_obs_, of an indicator is a weighted average of its protonated and deprotonated chemical shifts (δ_H_ and δ_L_, respectively), the pH of the solution is related to the p*K*_a_ of the indicator molecules through eq 4(16)
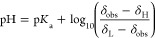
4where p*K*_a_ is of the “mixed” type.^27^ The pH was observed to drop from *ca*. 7.1 to *ca*. 5.1 for 24 h following inoculation, with the pH being higher at the colonocyte level compared to the mucin and commensal levels during the initial 16 h of the experiment (Figure 2a).

To probe the origin of the pH gradient across the depth of the NMR tube, we carried out metabolic profiling against a set of SCFAs—acetate, formate, succinate, lactate, propionate, and butyrate at the top (25–35 mm from the base of the NMR tube), middle (15–25 mm), and bottom (5–15 mm) regions of the sample. There were no statistically significant differences (*p* > 0.05) in the concentration of acetate, succinate, lactate, and formate among the top, middle, and bottom regions of the NMR tube (Figures S9 and S10, SI). However, the concentrations of butyrate and propionate were higher at the top than at the bottom (Figure 2b).

**Figure 3:**
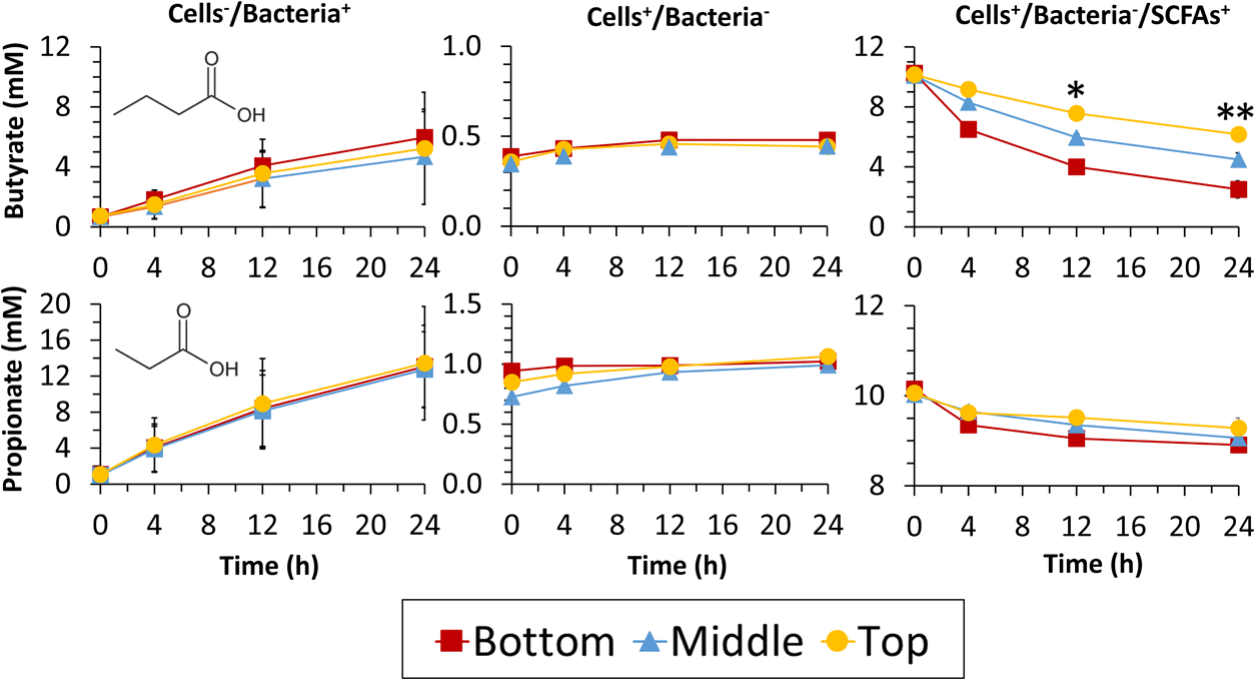
Concentration of butyrate and propionate over 24 h across the top (yellow circle, 25–35 mm), middle (blue triangle, 15–25 mm), and bottom (red square, 5–15 mm) of the NMR tube with fixed colonocytes and viable bacteria (left column), viable colonocytes and no bacteria (middle column), and viable colonocytes, no bacteria and media supplemented with propionate and butyrate (right column), *p < 0.05, **p < 0.01, *n* = 3.

To probe whether the changes in SCFA concentration at different depths were driven by the commensal bacteria, colonocyte metabolism, or a combination of both, three sets of controls were carried out (Figures 3 and S9, SI). There were no significant differences in the concentrations of propionate and butyrate at different depths when colonocytes were fixed (cells^–^/bacteria^+^; Figure 3, left). There was no SCFA production when commensals were filtered out prior to inoculation (cells^+^/bacteria^–^; Figure 3, middle). When bacteria were filtered out and propionate and butyrate were supplemented in the media, the concentration of both propionate and butyrate decreased the fastest nearer the colonocytes (Figure 3, right) compared to the middle and top regions of the NMR tube. These data suggest that colonocytes preferentially utilize butyrate and propionate over other SCFAs produced by commensal bacteria, which is also likely to drive the higher pH at the colonocyte level. Together, these data highlight the importance of applying spatially resolved NMR techniques for mapping out distance-dependent dynamic molecular exchange in heterogeneous systems featuring multiple cell types, providing details that would be missed by conventional nonlocalized analysis.

**Figure 4:**
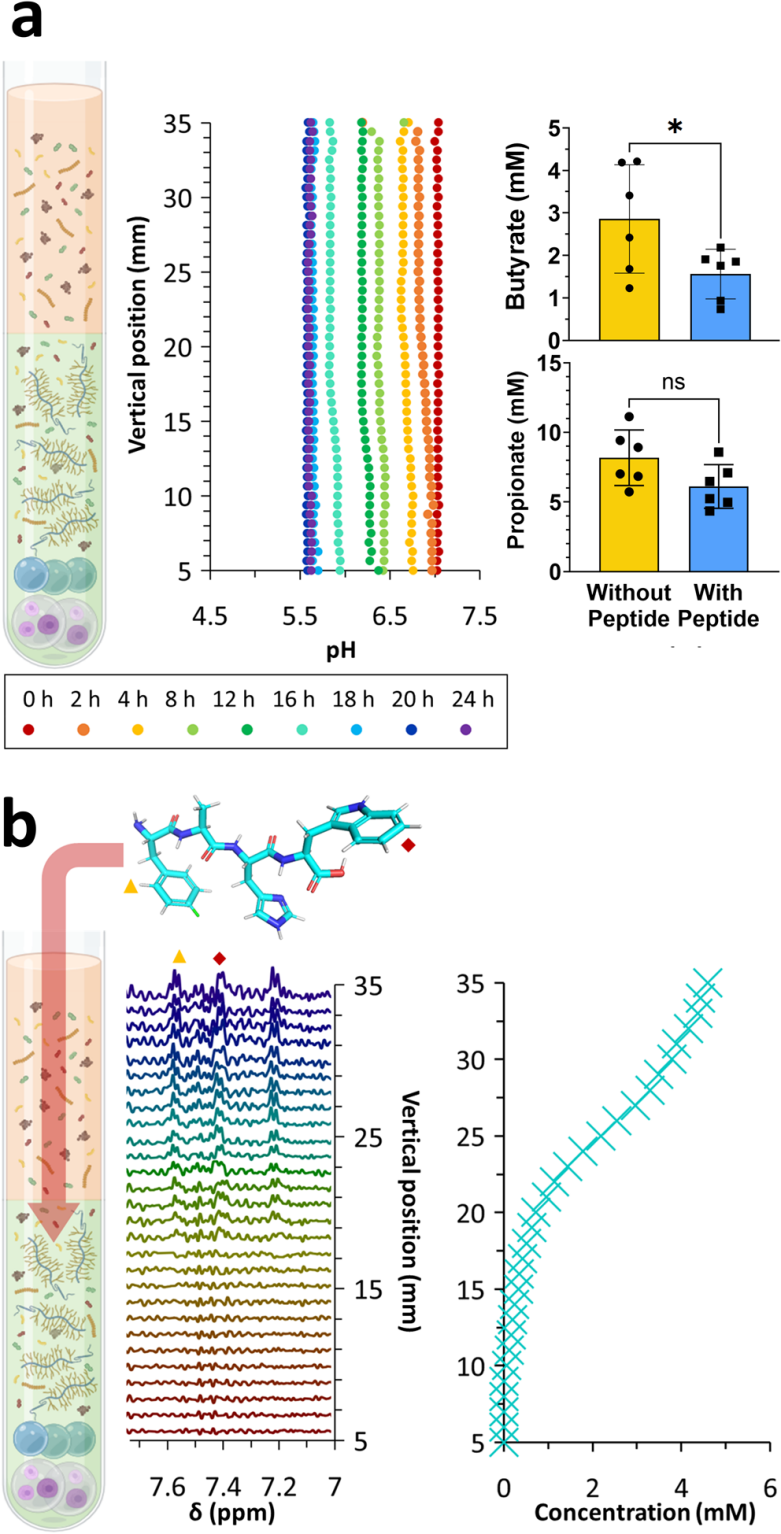
Spatially resolved pH change across our model over 24 h was observed with the introduction of a tetrapeptide. The average concentration of propionate and butyrate across the NMR tube after 24 h of fermentation with (blue) and without (yellow) the introduction of a fluorinated tetrapeptide in the media *p < 0.05, *n* = 3 (a). ^1^H chemical shift image (aromatic region) 6 h after the introduction of a fluorinated tetrapeptide at the top of the solution, with peak assignments of Phe (yellow triangle) and Trp (red diamond). Concentration mapping of the peptide along the depth of the NMR tube 6 h after peptide introduction (b), *n* = 3. Partially created in BioRender. Koev, T. (2025) https://BioRender.com/j03d772.

To assess the applicability of the model to probe the effect of active pharmaceutical ingredients (APIs) and API analogues on colonocyte and bacterial metabolism, as well as diffusion of the drug through the mucosal layer, a fluorinated tetrapeptide (*p*-F-Phe-Ala-His-Trp) was pipetted on top of the solution (1 mg in 10 μL of media), following inoculation. Changes in pH across the 24 h period were less pronounced, with the pH across different depths of the NMR tube dropping from 7.1 to 5.6 (vs 7.1 to 5.1 without the peptide; Figure 1a vs 3a). A decreased production of both propionate and butyrate was observed in the presence of the tetrapeptide (Figure 4a). The tetrapeptide was shown to have no significant (*p* > 0.05) effect on colonocyte viability but was shown to decrease bacterial viability (Figures S6 and S7, SI). The ability to trace and predict the diffusion of the tetrapeptide from the top of the NMR tube (intraluminal space) through the mucosa (Figure 4b) and all the way down to the bottom of the tube highlights the ability of our methodology to study drug transit in complex environments where semisolid materials such as mucosa are present.

Fitting the concentration of the peptide as a function of the vertical position in the NMR tube and time returns a diffusion coefficient in good agreement with the value predicted for water based on the molecular weight of the peptide tube (4.3 × 10^–10^ and 4.8 × 10^–10^ m^2^ s^–1^, respectively; Figures S4–S5 and S11, SI). These values suggest that the diffusion and local molecular mobility (NMR line width) of the peptide are not significantly affected by the mucosal layer. The low concentration of the peptide (<5 mM at any one point) is not expected to affect the pH of the sample.

## Conclusions

We demonstrate an easy-to-set-up model of the human large intestine, incorporating four colonocyte/enterocyte cell lines, tight and loosely bound mucosal layers, and fecal inoculum from a healthy volunteer. We have successfully shown how the model can capture the dynamic crosstalk between colonocytes and commensal bacteria using short (<10 min) CSI experiments, potentially aiding the development and screening of future targeted colonic drug delivery systems and APIs. The setup can be inoculated with an individual’s stool and harvested colonocytes, potentially enabling the development of personalized medicine for colorectal pathologies. Our CSI approach can be applied to any microbiological system involving immobilized and/or planktonic cells where concentration gradients are naturally present, for example, in microbial biofilms.^28^

## Data Availability

Raw NMR data sets will be available at: https://research-portal.uea.ac.uk/en/datasets/.

## Supporting Information Available

Ethical approval, spatially selective peptide diffusion, widefield and confocal microscopy, peptide diffusion modeling, full short-chain fatty acid panel, as well as metabolite NMR peak assignment (PDF)
