# Supplementary material for: Gut in Tube—Continuous Measurement of Metabolic Crosstalk between Cell Populations in Heterogeneous Samples by NMR Imaging
Source: Anal Chem. 2025 Feb 27;97(9):4962–8. doi: 10.1021/acs.analchem.4c05156 (PMC11912122; doi:10.1021/acs.analchem.4c05156)
Supplement: Supplementary file 1 — ac4c05156_si_001.pdf [file ac4c05156_si_001.pdf]

SUPPORTING INFORMATION FOR:

GUT IN TUBE – CONTINUOUS MEASUREMENT OF METABOLIC CROSSTALK BETWEEN CELL POPULATIONS IN HETEROGENEOUS SAMPLES BY NMR IMAGING

Todor T. Koev*, Hou Hei Chung, Caitlin Wright, Evie Banister, Stephen D. Robinson, Matthew Wallace*

t.koev@uea.ac.uk

matthew.wallace@uea.ac.uk

Table of Contents

1. Supplementary figures 2

2. Ethical approval for use of human tissue samples 9

1. Supplementary figures

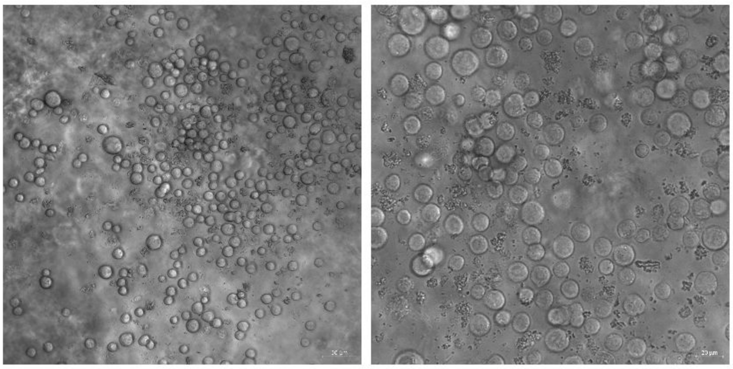

Figure S1. Brightfield microscopy of cells in alginate beads fresh after seeding (0 h).

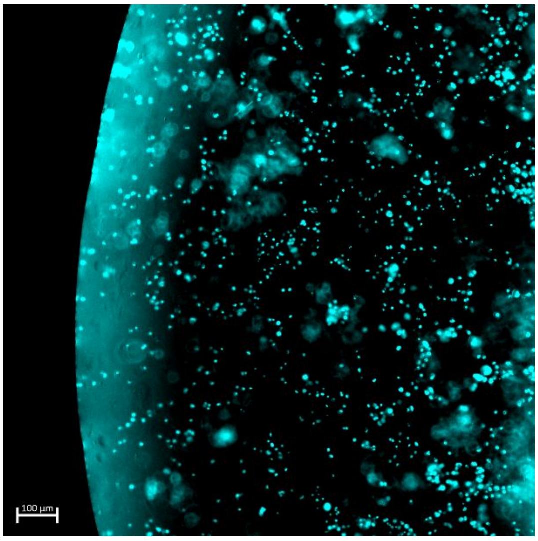

Figure S2. Fluorescence microscopy of all four cell lines (Caco-2, T84, SW620, HT29) in alginate beads fresh after seeding (0 h). Cellular DNA stained with Hoechst 33342.

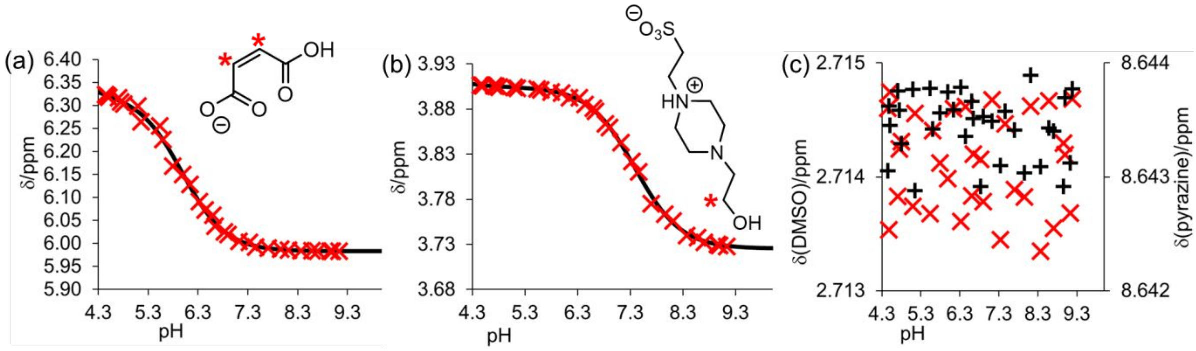

Figure S3. Plot of ^{1}H chemical shift of (a) maleate, (b) Hepes and (c) DMSO (red cross) and pyrazine (black vertical cross) versus pH, measured along pH gradient at 310 K. Black lines are fits to Equation S1.

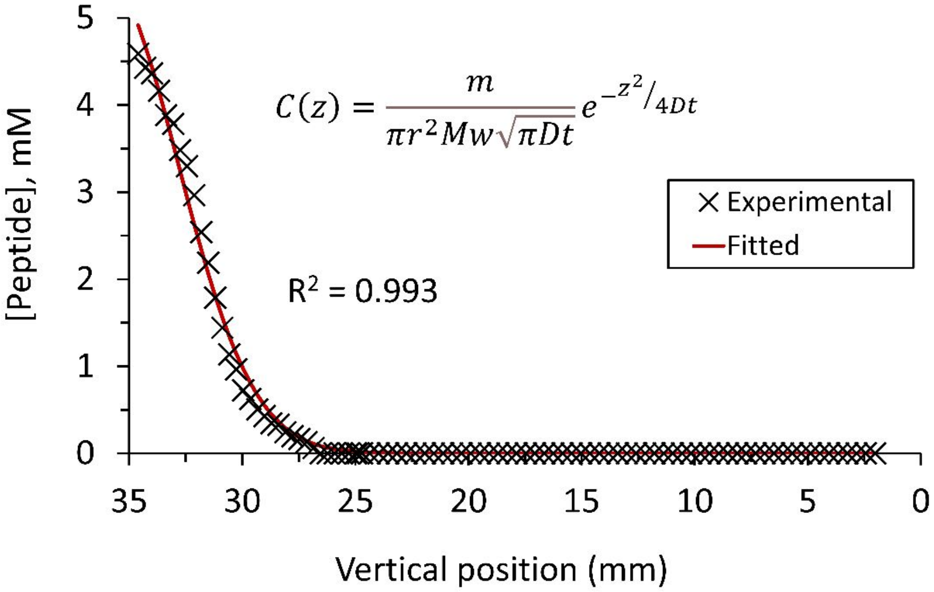

Figure S4. Experimentally measured (black cross) and mathematically fitted (red line) concentration of the peptide along the depth of NMR tube, 6 hours after preparation of the sample. D = 4.4x10^{-10} m^2s^{-1}

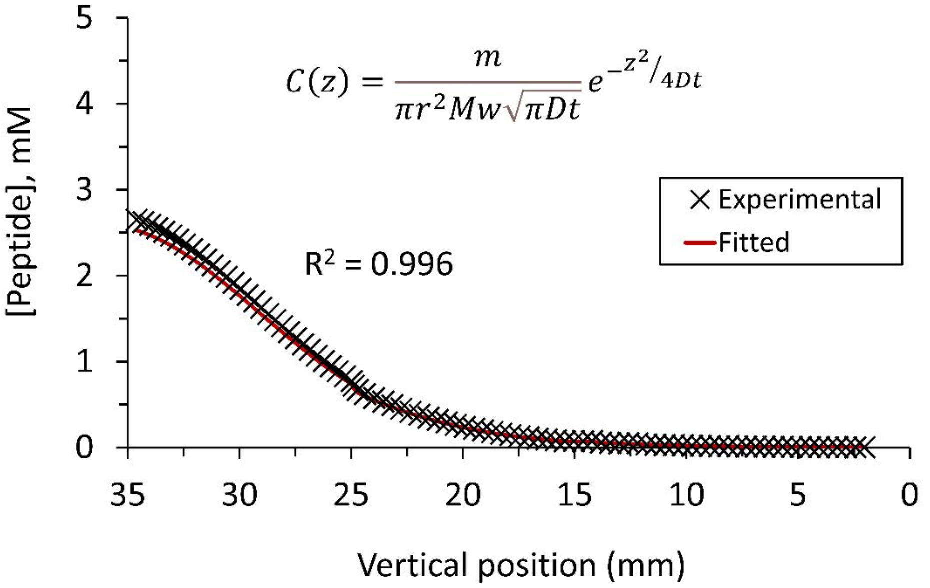

Figure S5. Experimentally measured (black cross) and mathematically fitted (red line) concentration of the peptide along the depth of NMR tube, 12 hours after preparation of the sample. D = 4.2x10^{-10} m^2s^{-1}

The self-diffusion coefficient of the peptide was calculated as 4.8x10^{-10} m^2s^{-1} using the Stokes-Einstein Gierer-Wirtz estimation (SEGWE).

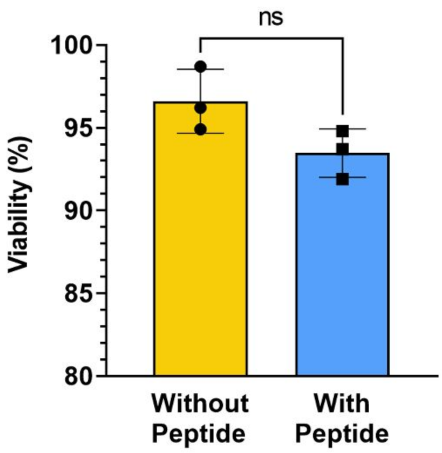

Figure S6. Colonocyte viability in the presence of a fluorinated tetrapeptide *p*-F-Phe-Ala-His-Trp, not statistically significant (ns), $p>0.05$, n=3.

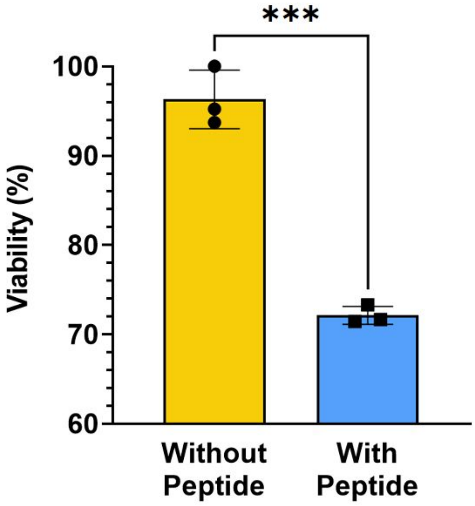

Figure S7. Bacterial viability in the presence of a fluorinated tetrapeptide *p*-F-Phe-Ala-His-Trp, ***$p<0.001$, n=3.

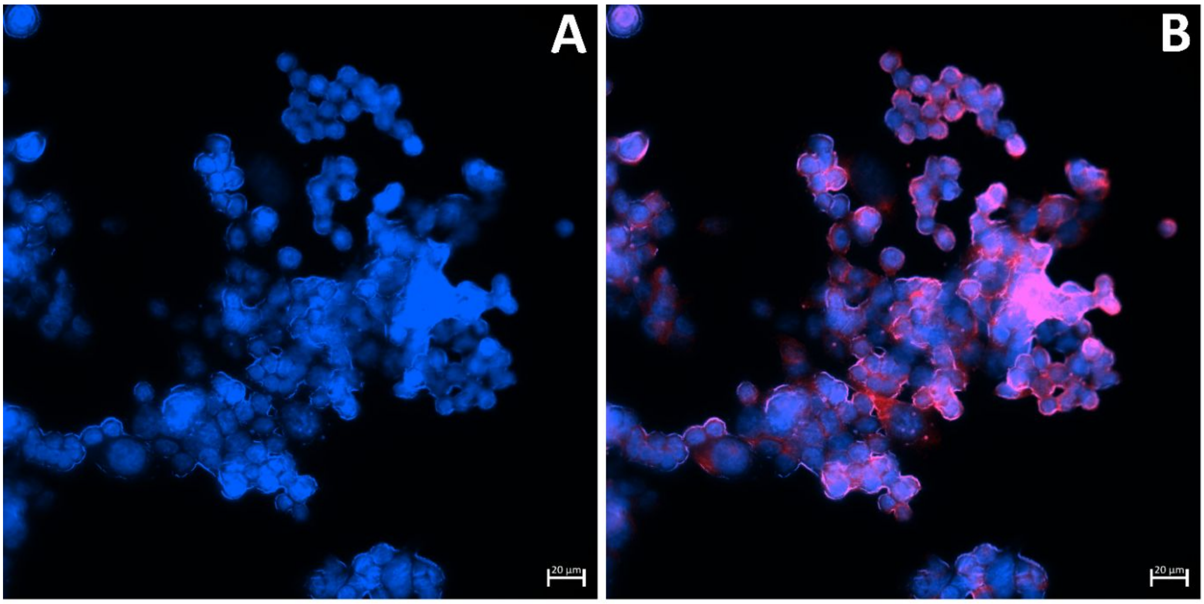

Figure S8. Fluorescence microscopy of all four cell lines (Caco-2, T84, SW620, HT29) forming multi-cellular clusters. Cellular DNA stained in blue (A), and surface (tightly bound) mucin (MUC2) stained in red (A, B).

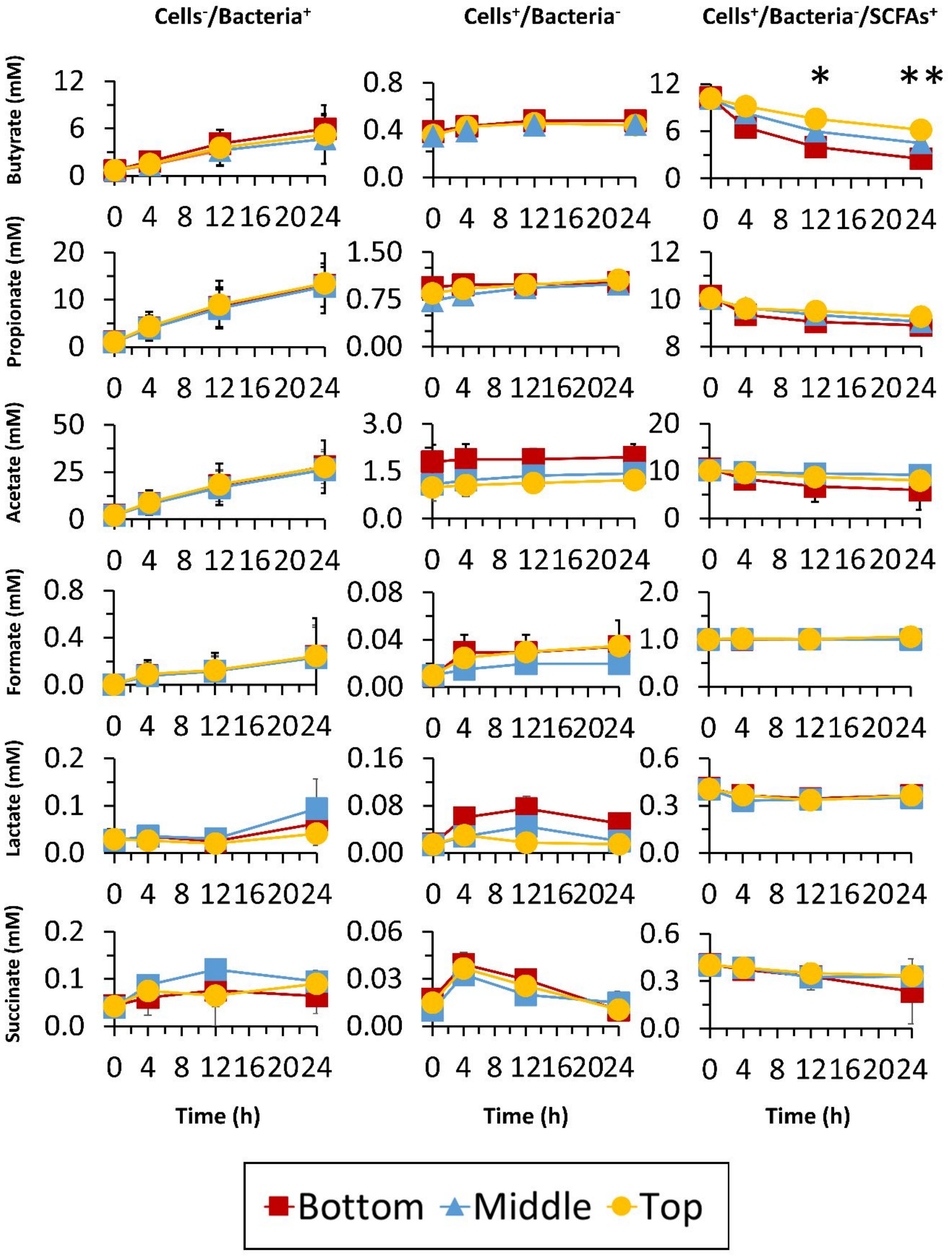

Figure S9. Concentration of butyrate, propionate, acetate, lactate, succinate and formate over 24 h across the top (yellow circle, 25-35 mm), middle (blue triangle, 15-25 mm) and bottom (red square, 5-15 mm) of the NMR tube with fixed colonocytes and viable bacteria (left column), viable colonocytes and no bacteria (middle column), and viable colonocytes, no bacteria and media supplemented with SCFAs (right column), * p<0.05, ** p<0.01, n=3.

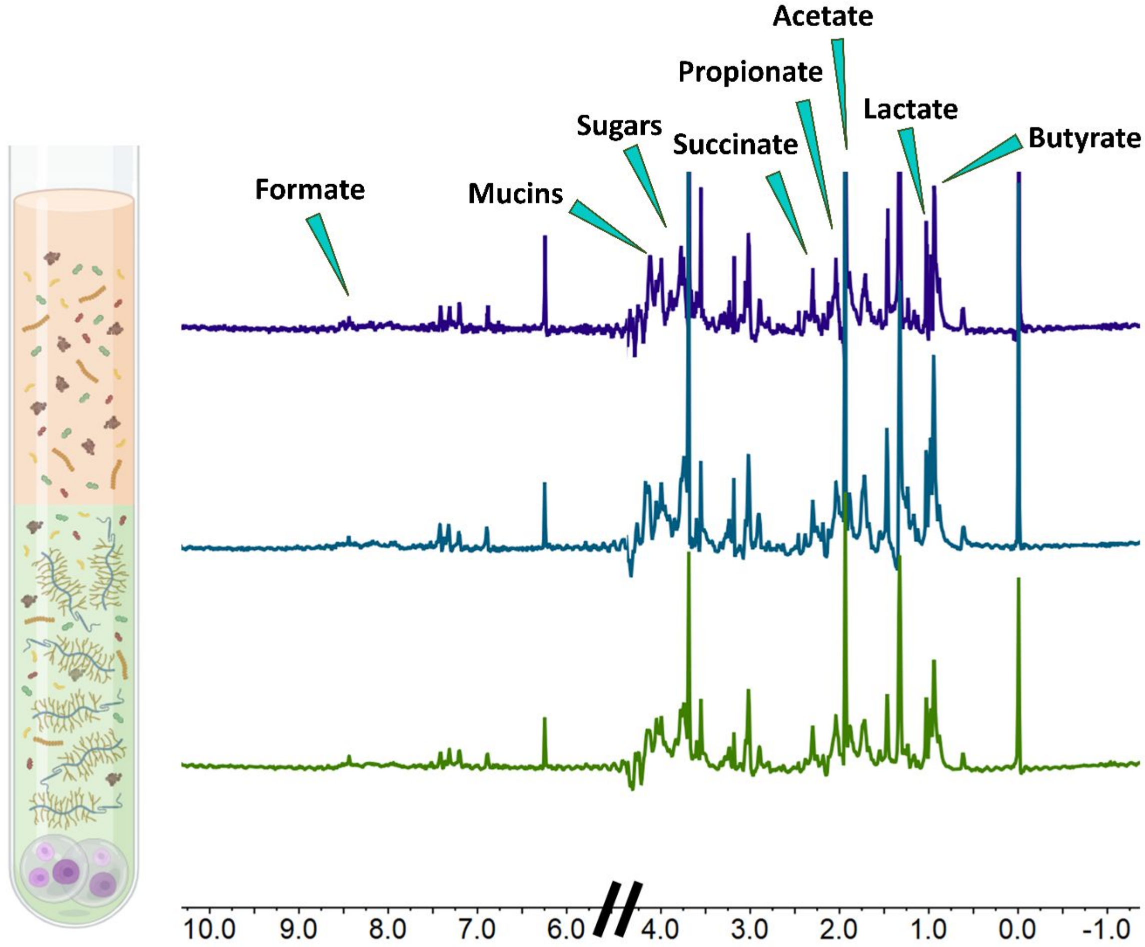

Figure S10. Superimposed slices from three different depths of the NMR tube at the end (24 h) of the fermentation experiment, with key species' peaks assigned. Partially *created in BioRender. Koev, T. (2025)* [*https://BioRender.com/j03d772*](https://BioRender.com/j03d772)

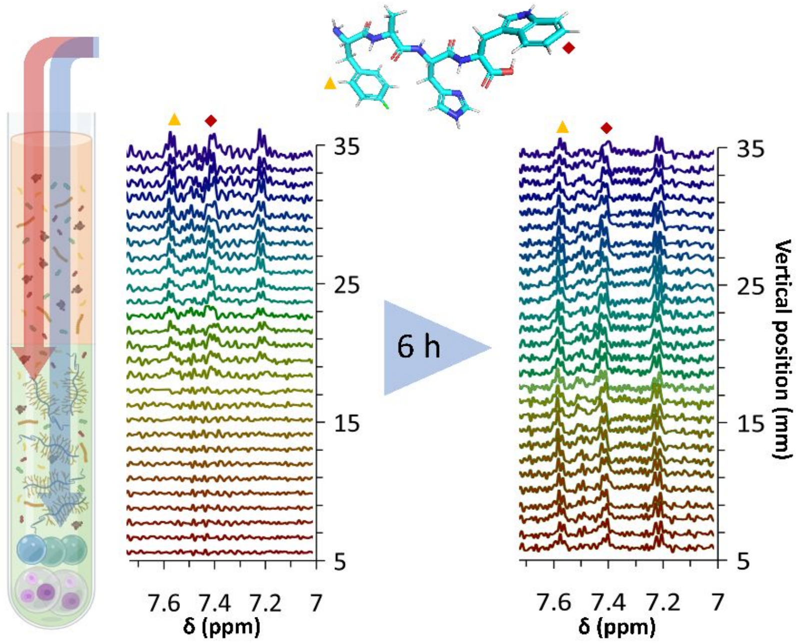

Figure S11. Chemical shift image of *in vitro* model 6 and 12 h (left and right, respectively) after the introduction of a fluorinated tetrapeptide at the top of the solution, zoomed in on the aromatic region (7-7.6 ppm) with peak assignment of Phe (yellow triangle) and Trp (red diamond). Diffusion of the tetrapeptide through the mucosal layer is essentially complete after 12 h. Partially *created in BioRender. Koev, T. (2025)* https://BioRender.com/j03d772

Table S1. δ_H, δ_L and pK_a for determination of pH in cell culture media at 310 K (I = 0.174 M) using Equation 1. We note that only acetate and maleate were used in this work, however data for other indicators is presented for convenience. All chemical shifts are reported relative to DSS (0.0 ppm).

**Indicator**	**δ_H/ppm**	**δ_L/ppm**	**pK_a^***	**I*/M**	**p$K_{a,0}$**	**pK_a (I = 0.174 M)**
Formate	8.2325	8.4437	3.57	0.05 (50 mM phthalate buffer)	3.66	3.52
Acetate	2.0856	1.9033	4.63	0.05 (50 mM phthalate buffer)	4.72	4.58
Maleate	6.3233	5.9833	5.98	0.11 M (pH gradient)	6.34	5.92
Hepes	3.9042	3.7252	7.38	0.11 M (pH gradient)	7.50	7.36
Methylphosphonate	1.2844	1.0711	7.60	0.1 M (25 mM K_2HPO_4 and 25 mM NaH_2PO_4)	7.95	7.53

2. Ethical approval for use of human tissue samples

A faecal sample was collected from one healthy individual. Ethical approval for the study was granted by the Human Research Governance Committee Norfolk and Norwich Biorepository (BAC.008.22, 23BR336) and the London - Westminster Research Ethics Committee (15/LO/2169).
